# Identification of novel components of the retinal determination gene network in *Drosophila* cell lines

**DOI:** 10.1098/rsob.250012

**Published:** 2025-07-16

**Authors:** Robert A. Drewell, Jacqueline M. Dresch

**Affiliations:** ^1^ Department of Biology, Clark University, Worcester, MA, USA

**Keywords:** *Drosophila*, *twin of eyeless*, *eyeless*, gene network, retinal determination

## Abstract

The retinal determination genetic network controls the development of the visual system in all seeing animals through the molecular regulation of cells to adopt an eye tissue fate. The compound eye of the fruit fly, *Drosophila melanogaster*, is an excellent model system to study the complex mechanisms within the network that regulate specification of cellular identity during embryogenesis. In *Drosophila*, the two *Pax6* paralogues, *eyeless* and *twin of eyeless*, sit at the very pinnacle of the network and their expression early in development activates critical downstream components of the retinal determination pathway. In this study, we investigate the expression of 21 known components of the network in two established embryonic cell lines, Kc167 and S2 cells, that show reciprocal expression patterns for the two *Pax6* paralogues. Network mapping reveals that many of the components of the network demonstrate extensive interactions with additional factors. Integrating the transcriptional profile of the cell lines, interaction maps and embryonic expression patterns enables us to identify 16 potential novel components of the genetic network, 11 of which are transcription factors. We confirm the regulatory potential for a subset of the novel transcription factors through the identification of predicted binding sites in previously characterized enhancers for the core genes in the network.

**Figure:**
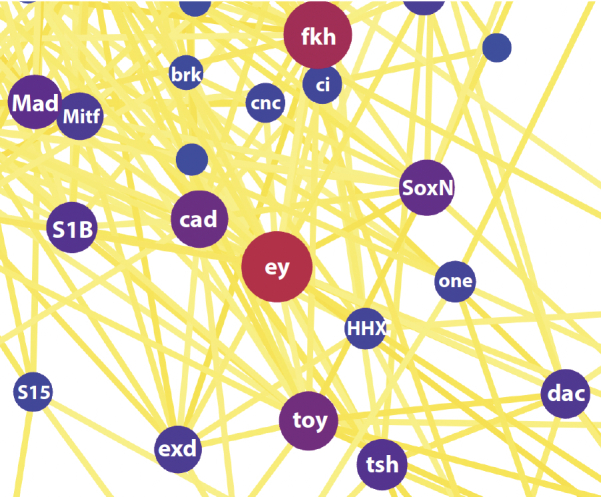

## 1. Introduction

The development of the eye relies on the molecular control of cellular specification in the embryo. Over the past 25 years, the evolutionarily conserved network that regulates this process in all seeing animals has been extensively characterized. This retinal determination gene network (RDGN) is now known to control differentiation, proliferation and specification of cell fates in a coordinated system that is not only essential to eye organogenesis [1,2], but is also important in tumour formation and progression in humans [3]. Many of the components representing the gene families in the RDGN were initially identified in *Drosophila*, including the founding member of the network, *eyeless*, discovered through mutation 100 years ago based on the no-eye phenotype of the flies [4]. The *eyeless* gene has a human homologue, *Pax6*, and encodes a PAX transcription factor with paired and homeodomain DNA-binding domains. Ectopic expression of *eyeless* can force cell populations in non-retinal tissues to adopt an eye fate [5] through the regulation of a large number of downstream target genes [6–8]. Functional orthologues of this master regulator have now been identified in nearly every phylum of the animal kingdom [1,9].

**Figure 1.**
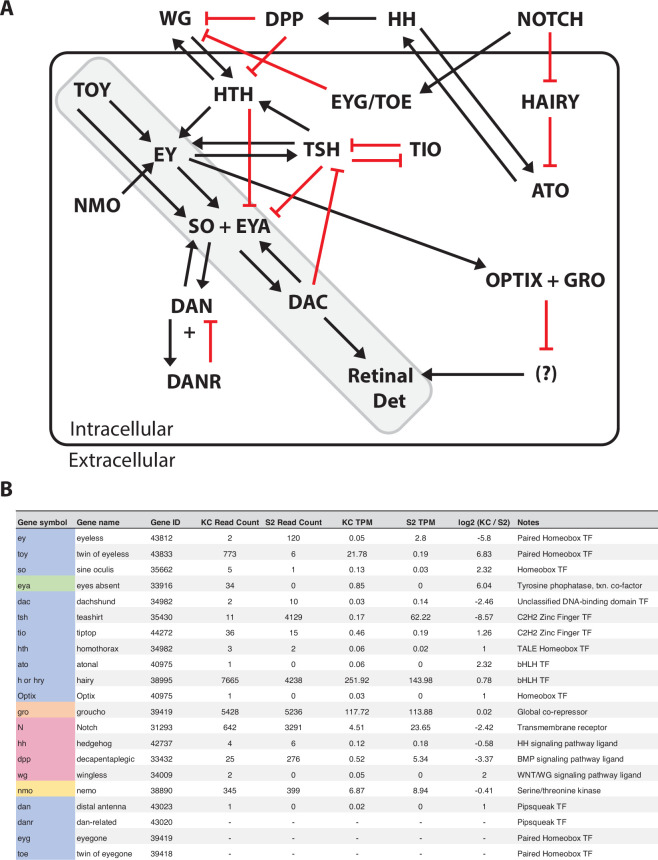
Retinal determination gene network. (A) *Drosophila* retinal determination gene regulatory network. An expanded RDGN is shown with 21 known components and all positive (activation, black) and negative (repression, red) interactions within the network. The core RD pathway is highlighted (shaded grey box). Note that the network contains autoregulatory circuits and feedback loops and includes extracellular signalling components. (B) Expression of known RDGN components in *Drosophila* embryonic cell lines. The expression of all 21 known components in Kc and S2 cells is listed with the read count, TPM values and log_2_ expression ratio for the two cell types. The functional activity of the protein encoded by each gene is also shown and colour coded: transcription factor (TF, blue), transcriptional co-factor (green), co-repressor (orange), protein kinase (yellow), extracellular signalling molecule (pink).

In *Drosophila*, *eyeless* (*ey*) and the closely related *Pax6* paralogue *twin of eyeless* (*toy*) sit at the very pinnacle of the RDGN [10–12]. Previous studies have shown that expression of *toy* is first detectable in embryos at cellular blastoderm stage 5 in an anterior dorsal band in the procephalic neuroectoderm [11,13], which eventually gives rise to the primordia of the visual system and brain [14]. By contrast, *ey* is first expressed during stage 10 (late germband extension) [11]. In addition, the TOY protein has been shown to directly activate the expression of *ey* through binding to an eye-specific enhancer [11,15,16]. These two PAX6 proteins then subsequently activate the transcription of several other downstream genes in the RDGN either independently or in combination, depending on the context of the target gene. Originally, 14 different genes were considered to be *bona fide* components of the RDGN based on their strong phenotypic effects when mutated [1,10]. Over time, that core list has been expanded as it has become clear that additional molecular components, including extracellular signalling pathways, play critical roles that impact the regulation of the network through biochemical, molecular or genetic interaction with existing members of the RD pathway (figure 1) [2].

*Drosophila* cell lines have been extensively used in wide-ranging studies of different biological processes [17,18]. Two of the most widely studied cell lines are Kc167 (Kc) [19] and Schneider 2 (S2) [20] cells, both of which were originally isolated from *Drosophila* embryos. Intriguingly, in our recent studies aimed at characterizing the global transcriptional profile in these cell types we discovered a strong reciprocal expression pattern for the two *Pax6* paralogues [21,22]. In Kc cells, there is a very high level of expression of *toy* (21.78 TPM) and virtually no expression of *ey* (0.05 TPM), while in S2 cells there is a high level of *ey* (2.8 TPM) and a very low level of *toy* (0.19 TPM) (table 1). These differences are not only statistically significant, but when compared with all 493 transcription factor-encoding genes expressed in the genome the magnitude of the fold change in expression between the two cell types (log_2_ Kc/S2) ranks *ey* as seventh out of the 66 genes significantly downregulated and *toy* as seventh out of the 57 genes significantly upregulated [22].

**Table 1.** Reciprocal expression profile for *eyeless* and *twin of eyeless* in *Drosophila* embryonic cell lines. The expression of the two *Drosophila Pax6* paralogues in Kc and S2 cells is listed with the read count, TPM values and log_2_ expression ratio for the two cell types. The fold change in expression for *eyeless* ranks it seventh out of the 66 TF genes that are expressed at significantly higher levels in S2 cells than Kc cells (Kc down). The fold change in expression for *twin of eyeless* ranks it seventh out of the 57 TF genes that are expressed at significantly higher levels in Kc cells than S2 cells (Kc up).

gene	symbol	gene ID	KC read count	S2 read count	fold difference	KC TPM	S2 TPM	log2 (KC/S2)	rank
*eyeless*	ey	43812	2	120	60	0.05	2.8	−5.8	7/66 down
*twin of eyeless*	toy	43833	773	6	128.83	21.78	0.19	6.83	7/ 57 up

The reciprocal transcriptional status of the *toy* and *ey* genes in the two cell types may therefore present a unique opportunity to investigate both the upstream control of their expression and the downstream impact on the RDGN. Neither cell type displays the phenotypic properties of an eye-like cell, despite expressing either *toy* (Kc) or *ey* (S2) at high levels, but rather appear to be of a haematopoietic origin [17,21,22]. We reasoned that this should enable us to examine the repressive components of the gene regulatory network that presumably prevent activation of the RD pathway in these cells and fully explore the transcriptional landscape to potentially identify additional, previously unknown, components of the RDGN.

## 2. Results and discussion

### 2.1. Retinal determination gene regulatory network in S2 and Kc cells

We conducted an extensive review of the literature to curate a comprehensive list of 21 molecular components of the retinal determination gene network (RDGN) that reflects the current understanding of the extended regulatory connections within the network (figure 1A). As might be expected in a regulatory network a majority (14) of the genes encode transcription factors (TFs), four genes encode extracellular signalling molecules (*Notch* (*N*), *hedgehog* (*hh*), *decapentaplegic* (*dpp*) and *wingless* (*wg*)), and the other three genes encode a transcriptional co-factor (*eyes absent* (*eya*)), a universal co-repressor (*groucho* (*gro*)) and a protein kinase (*nemo* (*nmo*)) (figure 1B). To assess whether the logic of the previously characterized regulatory interactions between these molecular components was maintained in the Kc and S2 cell lines, we examined the transcription of all 21 genes in the expanded RDGN.

In S2 cells, the *ey* gene is active (2.8 TPM) despite the absence of the TOY TF. Among the other known activators of *ey*, *homothorax* (*hth*) is not expressed, but *teashirt* (*tsh*) and *nmo* are present at high levels. In the case of *tsh*, the expression is specific to S2 cells (62.22 TPM), with very low levels of expression in Kc cells (0.17 TPM) correlating with the absence of *ey* transcription. The major downstream components of the core retinal determination pathway (*eya*, *sine oculis* (*so*) and *dachshund* (*dac*)) are all largely silenced in S2 cells, as might be predicted given that this cell type does not display an overall eye-like phenotype. The failure of EY to activate expression of *so* and *eya* in S2 cells could be partially explained by a combination of the absence of the known additional activators *distal antenna* (*dan*) and *distal antenna-related* (*danr*), along with the high level of TSH-mediated repression (figure 1).

In Kc cells, the high level of TOY (21.78 TPM) is not sufficient to activate *ey*, but it does appear to independently drive expression of *so* (0.13 TPM) and *eya* (0.85 TPM), as has been previously shown [23,24], albeit at relatively low levels. Expression of the *so* and *eya* genes may be facilitated by the much lower level of TSH, and its associated repressive activity, found in Kc cells when compared with S2 cells. However, as was the case in S2 cells, there is no downstream activation of *dac* in the Kc cells and therefore ultimately the cells do not follow a retinal determination fate (figure 1). In addition to the cell type-specific interactions we observed, some general features of the RDGN were uniformly reproduced in both cell types. The *wg*/*hth* and *hh*/*atonal* (*ato*) activation feedback loops were off in both cell types. By contrast, very high levels of *hairy* (*hry*), *gro* and *N* were detected in both cell types, which may be indicative of these well-characterized proteins playing important roles in a number of different regulatory pathways. Finally, *Optix* was not expressed in either cell type, suggesting that the characterized OPTIX-mediated parallel pathway to specify retinal determination [25,26] is also repressed in Kc and S2 cells. Taken as a whole, our transcriptome results therefore indicate that many, but not all, of the previously characterized regulatory interactions in the RDGN are reconstituted in the two cell types. Given that neither cell type displays overall neural phenotypic properties, but instead appear to be hematopoietic in origin [17,21,22], it is possible that the absence of expression for some of the RDGN components and their associated interactions may simply reflect the precise location and time in development at which these cells originated. A detailed investigation of the results will aid in furthering our understanding of previously characterized regulatory interactions as well as potentially identifying novel interactions within the RDGN.

### 2.2. Protein–protein interaction networks

**Figure 2.**
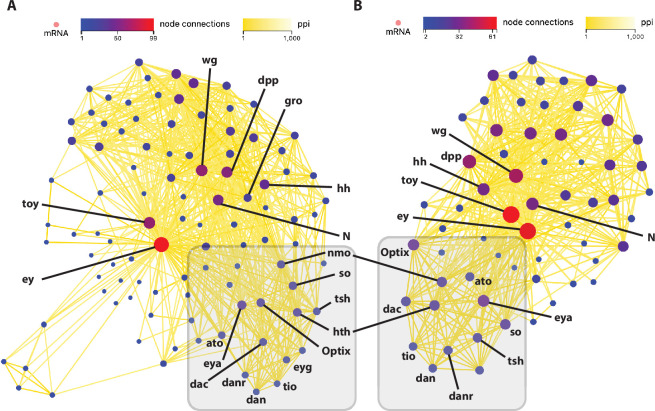
Protein–protein interaction network analyses. PPI network map for *eyeless* (A) and *twin of eyeless* (B). Circles indicate individual protein coding genes (nodes) and are colour coded according to the number of total connections from each node as indicated in the node connections colour code key. The line colour connecting the nodes indicates the relative strength of the calculated PPI value as shown in the ppi colour key. Individual genes known to be in the RDGN are labelled, with many demonstrating high connectivity in a sub-cluster within each network (shaded grey box).

To further explore the functional components of the RDGN and their potential regulatory interactions in the *Drosophila* cell lines, we performed protein–protein interaction (PPI) analysis. The network maps for the *ey* (figure 2A) and *toy* (figure 2B) genes both demonstrate considerable interaction. In the case of *ey*, there are 99 different node connections in the network representing the 99 genes that are found to have a direct connection to *ey*. For the 99 genes with connections, the total number of connections for each gene ranges from 1 to 58, with *toy* demonstrating the highest level of connectivity (figure 2A). Seventeen of the 99 genes (17.2%) have significantly lower expression in KC cells when compared to S2 cells (log_2_ Kc/S2 <−2), while 21 genes (21.2%) have significantly higher expression in KC cells when compared to S2 cells (log_2_ Kc/S2 > 2). In addition, 19 of the 21 known components of the RDGN are identified, with only *twin of eyegone* (*toe*) and *hry* absent (table 2).

In the *toy* network, there are 61 different node connections representing the 61 genes that are found to have a direct connection to *toy*. Among these 61 genes, the total number of connections for each gene ranges from 1 to 58, with *ey* demonstrating the highest level of connectivity (figure 2B). Thirteen of the 61 genes (21.3%) have significantly lower expression in KC cells when compared to S2 cells (log_2_ Kc/S2 <−2), while 15 genes (24.6%) have significantly higher expression in KC cells when compared to S2 cells (log_2_ Kc/S2 > 2). Within the *toy* network, 17 of the 21 known components of the RDGN are identified, with *eyegone* (*eyg*), *toe*, *gro* and *hry* absent (table 3). Additionally, we also probed the interactions of *ey* and *toy* with non-coding RNAs in the *Drosophila* genome and identified 27 predicted interacting miRNAs for *ey* (electronic supplementary material, table S1) and 12 for *toy* (electronic supplementary material, table S2).

**Table 2.** Genes in the *eyeless* PPI network ranked by node connectivity. The 99 genes in the *eyeless* PPI network are listed with the number of node connections, the read count, TPM values and log_2_ expression ratio in Kc and S2 cell lines, and whether the gene is a known component of the RDGN. The two *Pax6* paralogues, *eyeless* and *twin of eyeless*, are highlighted in blue. Many of the genes demonstrate a significant fold change in expression between the two cell types. Genes that are expressed at much lower levels in Kc cells than S2 cells (log_2_ (Kc/S2) < −4) are highlighted in orange. Genes that are expressed at much higher levels in Kc cells than S2 cells (log_2_ (Kc/S2) > 4) are highlighted in green.

gene symbol	gene ID	node conn	KC TPM	S2 TPM	KC read count	S2 read count	log2 (KC/S2)	RDGN
'ey'	43812	99	0.05	2.8	2	120	−5.80	yes
'toy'	43833	58	21.78	0.19	773	6	6.83	yes
'wg'	34009	57	0.05	0	2	0	2.00	yes
'dpp'	33432	52	0.52	5.34	25	276	−3.37	yes
'N'	31293	47	4.51	23.65	642	3291	−2.42	yes
'Wnt5'	32838	40	24.58	16.25	1318	885	0.57	no
'hh'	42737	39	0.12	0.18	4	6	−0.58	yes
'Wnt4'	34007	38	0.02	0	1	0	1.00	no
'Wnt2'	35975	38						no
'eya'	33916	32	0.85	0	34	0	6.04	yes
'pan'	43769	30	21.99	25.51	1310	1750	−0.24	no
'fkh'	43383	29	0.15	0	8	0	3.58	no
'ect'	44135	27	0	0.06	0	2	−2.32	no
'hth'	41273	27	0.06	0.02	3	2	1.00	yes
'Optix'	44108	27	0.03	0	1	0	1.00	yes
’so'	35662	26	0.13	0.03	5	1	2.32	yes
'Sox100B'	45039	25	0.04	1.46	1	41	−5.27	no
'tin'	42536	24	0.22	0.18	5	4	0.28	no
'nmo'	38890	24	6.87	8.94	345	399	−0.41	yes
'gro'	43162	24	117.72	113.88	5428	5236	0.02	yes
'SoxN'	44275	23	0.77	0.05	43	3	3.91	no
'nau'	42799	23	0	0.43	0	9	−5.09	no
'nej'	43856	23	15.25	19.36	3176	4295	−0.37	no
'Mad'	33529	21	14.82	47.07	553	1775	−1.69	no
'bnl'	42356	21	45.35	1.23	2626	70	5.18	no
’shg'	37386	21	2.57	3.66	230	330	−0.54	no
'dac'	34982	21	0.03	0.14	2	10	−2.46	yes
'tsh'	35430	20	0.17	62.22	11	4129	−8.57	yes
'pnr'	44849	20	1.41	221.13	55	8652	−7.32	no
'byn'	39349	19						no
'ato'	40975	19	0.06	0	1	0	2.32	yes
'tup'	35147	18						no
'ci'	43767	17	1.69	0.3	116	22	2.46	no
'eyg'	39419	17						yes
'Decay'	42008	15	0.14	0	2	0	3.46	no
'danr'	43020	15						yes
'bi'	31379	15						no
’smo'	33196	15	8.34	13.02	464	730	−0.67	no
'exd'	32567	14	63.15	90.49	2160	3157	−0.55	no
'Six4'	40297	14						no
'Antp'	40835	14	0.04	0	2	0	1.58	no
'foxo'	41709	14	24.84	9.28	1276	486	1.39	no
'tio'	44272	13	0.46	0.19	36	15	1.26	yes
'dan'	43023	13	0.02	0	1	0	1.00	yes
'elav'	31000	13	2.87	5.43	210	307	−0.94	no
'CG10827'	42482	12						no
'tkv'	33753	12	8.24	16.1	325	640	−0.99	no
’slp1'	33607	12						no
'His3:CG33803'	3772149	12	0	4.19	0	27.22	−8.38	no
'Alp4'	43671	11	0.24	0.88	7	26	−1.88	no
'His3:CG33866'	3772189	11	0	0.84	0	5.44	−6.07	no
'Alp2'	37539	11	0.08	0	2	0	2.81	no
'CG1809'	35981	11	0.88	0	20	0	6.11	no
'upd1'	32813	10	0	0.03	0	1	−1.00	no
'phu'	41135	10	1.7	0.2	43	5	3.06	no
'CG3292'	37538	10	0.52	0	12	0	5.32	no
'da'	34413	10	40.23	87.24	1759	3851	−1.14	no
'CG3264'	37540	10	1.1	0	26	0	6.43	no
’slp2'	33608	9						no
'tll'	43656	9	0.04	0	1	0	1.58	no
'Poxm'	40990	8	0	0.11	0	4	−3.17	no
'Hr51'	36702	8	0.04	0.49	1	14	−3.70	no
'lz'	31883	8	0.02	5.14	1	240	−7.68	no
'D'	39570	8						no
'mirr'	39441	8	0.12	0.41	5	22	−1.83	no
'amos'	35110	8						no
'Sox15'	36575	7	16.51	0.12	835	6	7.16	no
'fng'	40314	7	1.72	1.15	46	31	0.55	no
'brk'	31665	7	25	11.74	1103	522	1.06	no
'acj6'	47080	7	0.14	0.07	7	4	1.14	no
'pb'	40826	7						no
'ple'	38746	6	0.02	0.12	1	5	−2.32	no
'ninaE'	42367	6						no
'Hipk'	38070	6	28.98	158.72	2969	16 826	−2.48	no
'Rh4'	39887	5	1.83	0	33	0	7.16	no
'Rh3'	42398	5	0.05	0	1	0	2.00	no
'Rh2'	42261	5	0	0.16	0	3	−3.70	no
'gish'	49701	5	129.05	137.26	5317	5794	−0.12	no
'Mitf'	3885647	5	11.15	20.06	486	884	−0.87	no
'dsx'	40940	5	0.02	0.02	1	1	0.00	no
'Syt1'	33473	5	0.02	0.04	2	3	−0.58	no
'tj'	35227	5	0	0.02	0	1	−1.00	no
'Bro'	38202	4	0.11	0	1	0	3.00	no
'Sox21a'	39567	4	0.06	0	2	0	2.32	no
'mew'	32275	3	12.92	61.5	885	4191	−2.28	no
'Cbp53E'	36905	3						no
'Sox14'	37822	3	13.97	19.06	574	827	−0.47	no
'Mst87F'	41693	2	3.51	0	20	0	8.10	no
'CG30324'	246540	2	0.25	0.85	2	7	−1.84	no
'CG7423'	32894	2						no
'CG31715'	318909	2	90.04	89.38	648	646	−0.02	no
'CG15771'	31500	2	58.03	77.62	1847	2461	−0.45	no
'Crys'	34604	2						no
'cdm'	42171	1	30.5	36.98	1388	1688	−0.30	no
'CG5011'	50196	1	0.19	1.14	1	6	−2.60	no
'Nf-YA'	39091	1	22.51	36.61	631.02	1033.66	−0.73	no
'AOX1'	41894	1	61	34.71	3582	2054	0.79	no
'CG42688'	10178824	1						no
'disco'	32579	1	0.02	0	1	0	1.00	no
'ct'	44540	1	20.85	28.35	3070	4201	−0.47	no

**Table 3.** Genes in the *twin of eyeless* PPI network ranked by node connectivity. The 61 genes in the *twin of eyeless* PPI network are listed with the number of node connections, the read count, TPM values and log_2_ expression ratio in Kc and S2 cell lines, and whether the gene is a known component of the RDGN. The two *Pax6* paralogues, *twin of eyeless* and *eyeless*, are highlighted in blue. Many of the genes demonstrate a significant fold change in expression between the two cell types. Genes that are expressed at much lower levels in Kc cells than S2 cells (log_2_ (Kc/S2) < −4) are highlighted in orange. Genes that are expressed at much higher levels in Kc cells than S2 cells (log_2_ (Kc/S2) > 4) are highlighted in green.

gene symbol	gene ID	node conn	KC TPM	S2 TPM	KC read count	S2 read count	log2 (KC/S2)	RDGN
'toy'	43833	61	21.78	0.19	773	6	6.83	yes
'ey'	43812	58	0.05	2.8	2	120	−5.80	yes
'wg'	34009	42	0.05	0	2	0	2.00	yes
'dpp'	33432	38	0.52	5.34	25	276	−3.37	yes
'Wnt5'	32838	32	24.58	16.25	1318	885	0.57	no
'N'	31293	31	4.51	23.65	642	3291	−2.42	yes
'Wnt4'	34007	30	0.02	0	1	0	1.00	no
'Wnt2'	35975	30						no
'hh'	42737	30	0.12	0.18	4	6	−0.58	yes
'cad'	35341	29	1.36	0.03	46	1	5.74	no
'eya'	33916	27	0.85	0	34	0	6.04	yes
'Sox100B'	45039	25	0.04	1.46	1	41	−5.27	no
'ect'	44135	25	0	0.06	0	2	−2.32	no
'Optix'	44108	25	0.03	0	1	0	1.00	yes
'fkh'	43383	23	0.15	0	8	0	3.58	no
'SoxN'	44275	22	0.77	0.05	43	3	3.91	no
'pan'	43769	22	21.99	25.51	1310	1750	−0.24	no
’so'	35662	21	0.13	0.03	5	1	2.32	yes
'bnl'	42356	20	45.35	1.23	2626	70	5.18	no
'hth'	41273	20	0.06	0.02	3	2	1.00	yes
'pnr'	44849	20	1.41	221.13	55	8652	−7.32	no
'nmo'	38890	19	6.87	8.94	345	399	−0.41	yes
'byn'	39349	19						no
'nau'	42799	19	0	0.43	0	9	−5.09	no
'dac'	34982	17	0.03	0.14	2	10	−2.46	yes
'tup'	35147	17	0	0	0	0		no
'tsh'	35430	16	0.17	62.22	11	4129	−8.57	yes
'CG10827'	42482	14						no
'vnd'	31003	14						no
'danr'	43020	14						yes
'ato'	40975	14	0.06	0	1	0	2.32	yes
'Alp4'	43671	13	0.24	0.88	7	26	−1.88	no
'Alp2'	37539	13	0.08	0	2	0	2.81	no
'tio'	44272	12	0.46	0.19	36	15	1.26	yes
'phu'	41135	12	1.7	0.2	43	5	3.06	no
'CG3292'	37538	12	0.52	0	12	0	5.32	no
'Six4'	40297	12						no
'dan'	43023	12	0.02	0	1	0	1.00	yes
'CG1809'	35981	12	0.88	0	20	0	6.11	no
'CG3264'	37540	12	1.1	0	26	0	6.43	no
'Decay'	42008	11	0.14	0	2	0	3.46	no
'Antp'	40835	11	0.04	0	2	0	1.58	no
’smo'	33196	11	8.34	13.02	464	730	−0.67	no
'Sox15'	36575	8	16.51	0.12	835	6	7.16	no
'upd1'	32813	7	0	0.03	0	1	−1.00	no
'ple'	38746	7	0.02	0.12	1	5	−2.32	no
’slp1'	33607	7						no
’slp2'	33608	6						no
'Hipk'	38070	5	28.98	158.72	2969	16 826	−2.48	no
'acj6'	47080	5	0.14	0.07	7	4	1.14	no
'Mitf'	3885647	4	11.15	20.06	486	884	−0.87	no
'Syt1'	33473	4	0.02	0.04	2	3	−0.58	no
'tj'	35227	4	0	0.02	0	1	−1.00	no
'Hr51'	36702	3	0.04	0.49	1	14	−3.70	no
'Cbp53E'	36905	3						no
'Sox21a'	39567	3	0.06	0	2	0	2.32	no
'Sox14'	37822	3	13.97	19.06	574	827	−0.47	no
'CG7423'	32894	2						no
'mew'	32275	2	12.92	61.5	885	4191	−2.28	no
'Zif'	40795	2	45.82	49.13	900	967	−0.13	no
'CG31715'	318909	2	90.04	89.38	648	646	−0.02	no
'Crys'	34604	2						no

A large number of protein-coding genes not on the list of 21 known RD members are identified in the network maps for both *ey* (80 genes) and *toy* (44 genes), including many with equivalent levels of connectivity to the previously identified RDGN components. If we only consider genes with at least 14 node connections, in the *ey* PPI network there are 25 genes, 15 of which demonstrate an expression read count of 8 or above in at least one cell type (table 2). Using the same threshold criteria, in the *toy* PPI network there are 16 genes, 10 of which are expressed above the threshold level (table 3). Among these genes, nine are shared between the two networks: *Wnt oncogene analog 5* (*Wnt5*), *pangolin* (*pan*), *fork head* (*fkh*), *Sox100B*, *SoxNeuro* (*SoxN*), *nautilus* (*nau)*, *branchless* (*bnl*), *pannier* (*pnr*) and *smoothened* (*smo*). Only *caudal* (*cad*) is specific to the *toy* network, while *nejire* (*nej*), *Mothers against dpp* (*Mad*), *shotgun* (*shg*), *cubitus interruptus* (*ci*), *extradenticle* (*exd*) and *fork head box O* (foxo) are specific to the *ey* network. It should be noted that *Mad* is a known effector of the previously characterized *dpp* signalling molecule in the RDGN and therefore it is perhaps not unsurprising to find it identified in the *ey* PPI network. Likewise, *ci* is known to be a downstream effector of *hh* signalling.

**Figure 3.**
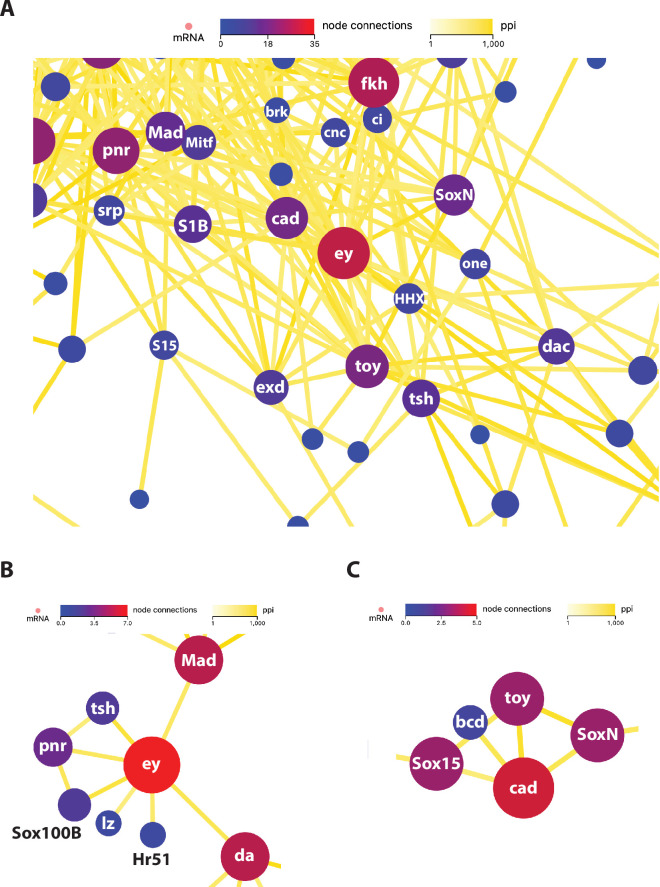
Protein–protein transcription factor interaction network. (A) PPI network map for all 493 TF genes expressed in *Drosophila* Kc or S2 cells. The sub-network map that contains *eyeless* and *twin of eyeless* is shown. Circles indicate individual protein coding genes (nodes) and are colour coded according to the number of total connections from each node as indicated in the node connections colour code key. The line colour connecting the nodes indicates the relative strength of the calculated PPI value as shown in the ppi colour key. Individual genes are labelled with standard abbreviations, except for S1B = *Sox100B*, S15 = *Sox15*, HHX = *HHEX*, one = *onecut*. (B) Interactions between the 66 TF genes that are expressed at significantly higher levels in S2 cells than Kc cells (Kc down), reveals a distinct sub-cluster containing *eyeless*. (C) Interactions between the 57 TF genes that are expressed at significantly higher levels in Kc cells than S2 cells (Kc up), reveals a distinct sub-cluster containing *twin of eyeless*.

As 11 of the 16 genes identified above are TFs, we expanded our analysis to examine the connectivity of the two *Pax6* paralogues in the PPI network consisting of all 493 TFs expressed in *Drosophila* Kc and/or S2 cells [22]. Not surprisingly, *ey* and *toy* are firmly embedded in the cluster of genes representing the known TF components in the RDGN and display extensive connectivity (figure 3A). Additionally, if we focus on the connectivity of *ey* among the 66 TF genes that are expressed at significantly lower levels in KC cells compared with S2 cells, then a distinct sub-cluster is identified that includes; *tsh*, *pnr*, *Sox100B*, *lozenge* (*lz*), *Hormone receptor 51* (*Hr51*), *daughterless* (*da*) and *Mad* (figure 3B). Likewise, if we restrict the analysis to the connectivity of *toy* among the 57 TF genes that are expressed at significantly higher levels in KC cells compared to S2 cells, another clear sub-cluster emerges that includes; *bicoid* (*bcd*), *cad*, *Sox box protein 15* (*Sox15*) and *SoxN* (figure 3C). Among all of these TF-encoding genes, only the maternally expressed *bcd* does not appear on our original PPI lists of 99 interacting genes for *eye* or 61 interacting genes for *toy*.

### 2.3. Known upstream *toy* interactors

Early studies in the Gehring lab were able to identify maternal and gap TFs involved in regulating the precise expression of *toy* in the early embryo [27]. The maternally deposited and anteriorly localized *bcd* mRNA, which is translated to produce a gradient of BCD TF along the anterior–posterior (AP) axis after fertilization, was found to activate *toy* expression in the anterior of the embryo. By contrast, the CAD gap TF plays a role in the repression of *toy* in the posterior of the embryo. Along the dorsal–ventral (DV) axis of the embryo, peak levels of DECAPENTAPLEGIC (DPP) are critical to restrict *toy* expression at the dorsal midline. These regulatory roles were confirmed in our own recent study of the control of *toy* transcription in the early embryo [13]. We therefore wanted to examine the expression profile of all three of these genes in the cell lines and explore their connection to *toy* regulation.

In Kc cells, *toy* is expressed at a high level (21.78 TPM in Kc, 0.19 TPM in S2) (table 1). It is therefore perhaps unsurprising to find *bcd* also expressed at a high level in Kc cells (4.84 TPM in Kc, 0 TPM in S2). Indeed, the activation from the presumably high level of BCD in the Kc cells appears to be strong enough to counter any repression from *cad*, which is also expressed in these cells, albeit at a lower level (1.36 TPM in Kc, 0.03 TPM in S2). Intriguingly, expression of *dpp* is relatively low in Kc cells when compared with S2 cells (0.52 TPM in Kc, 5.34 TPM in S2) supporting the possibility of a concentration-dependent role in the regulation of *toy* expression.

**Figure 4.**
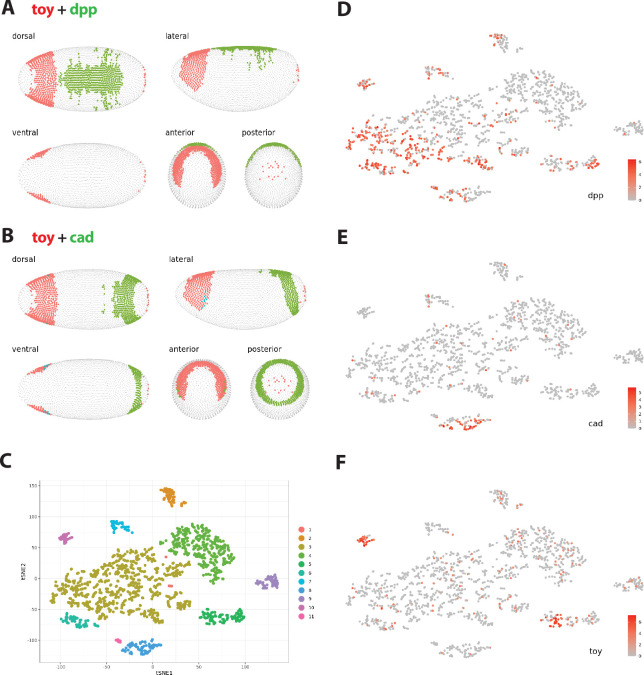
Embryonic expression and single-cell sequencing cluster identity of upstream RDGN components. (A) Expression of *twin of eyeless* (red) and *decapentaplegic* (green) in stage 6 embryos. (B) Expression of *twin of eyeless* (red) and *caudal* (green) in stage 6 embryos. (C) Two-dimensional t-SNE representation shows the 11 major clusters in stage 6 embryos grouped by transcriptome similarity. Expression of *decapentaplegic* (*dpp*) (D), *caudal* (*cad*) (E) and *twin of eyeless* (*toy*) (F), in t-SNE clustered cells are shown. All data generated from DVEX platform as described in §4.

The regulatory roles for DPP and CAD suggested by their transcriptional profiles in the two cell types are also supported by their expression patterns in embryos. In stage 6 embryos, *toy* expression is restricted to a precise anterior dorsal band, that is mutually exclusive with the *dpp* (centre of dorsal midline) and *cad* (posterior) domains of expression (figure 4A,B). Furthermore, if we examine the t-SNE representation of the eleven major cell clusters grouped by transcriptome similarity at this embryonic stage, *toy* (group 5 and 10) shows no overlap with *cad* (group 8) (figure 4C). The t-SNE profiles for *dpp* and *toy* do show some overlap, particularly in groups 5 and 10 (figure 4D). The low level of *dpp* transcription found in Kc cells, along with the modest overlap of *dpp* and *toy* t-SNE profiles, suggests that these cells may have originated from a more anterior region of the embryo (figure 4A). This result, once more, suggests that DPP may be regulating *toy* in a concentration-dependent manner, specifically that the high level of DPP found towards the posterior of the dorsal midline is repressive and that a lower level of DPP may potentially activate *toy* in the anterior of the embryo. As a maternally expressed gene, *bcd* is not transcribed in the embryo. Other gap genes previously shown to play a role in controlling *toy* transcription, including *hunchback* (*hb*), *knirps* (*kni*) and *dorsal* (*dl*) [13,27], are all either not expressed or expressed at very low levels (read count < 5) in both cell types, indicating that the upstream regulatory network responsible for *toy* activation in the embryo, with the exception of BCD, CAD and DPP, may be largely inactive in both cell types.

### 2.4. Regulatory interactions of known downstream RDGN components

Of the 21 previously characterized components of the RDGN (figure 1), 19 were identified in our PPI analysis, with only *hry* and *toe* absent (figure 2). Among the 19, all had a high level of connectivity in the *ey* and *toy* PPI networks (>12 node connections) (tables 2 and 3). Consistent with the fact that neither the Kc or S2 cells display eye-like phenotypic properties, 10 genes showed expression of 10 or less read counts in both cell types (*wg*, *hh, hth*, *Optix*, *so*, *dac*, *ato*, *eyg*, *danr* and *dan*). Despite the absence of these components of the RDGN, the nine other genes (*ey*, *toy, eya*, *tsh*, *tio*, *gro*, *N*, *dpp* and *nmo*) had high level expression in at least one cell type. Strikingly, only three (*tio*, *gro* and *nmo*) of the nine genes did not demonstrate a significant fold change in expression between the two cell types (log_2_ Kc/S2 <−2 or >2). Taken together, these results indicate that distinct elements of a truncated RDGN may well be functioning in the Kc and S2 cells.

**Figure 5.**
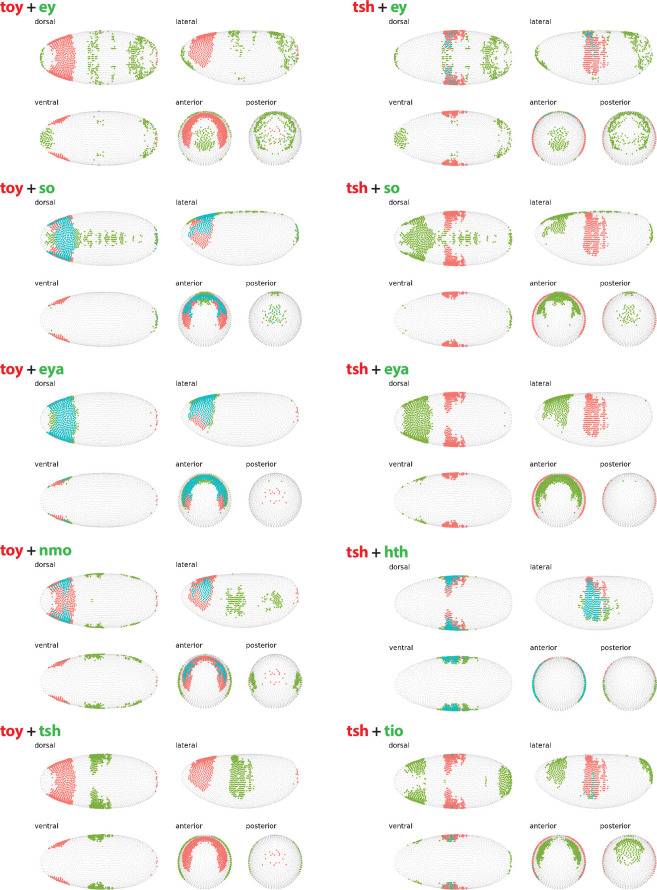
Embryonic expression patterns of select known RDGN components. The expression of two genes previously identified as a component of the RDGN is shown in each panel. The genes and their expression in stage 6 embryos are colour coded in red and green, with any overlapping expression shown in blue. All data generated from DVEX platform as described in §4.

To further address the functional relevance of the transcription of the RDGN components in the cells we also analysed their expression in embryos. Of the 21 known RDGN members, only *eyg* and *toe* do not have characterized expression patterns in stage 6 embryos [28], with the remaining 19 genes demonstrating diverse expression patterns (electronic supplementary material, figure S1). The two *Pax6* paralogues, *toy* and *ey*, are expressed in non-overlapping patterns in the embryo (figure 5), mirroring their reciprocal expression in Kc and S2 cells, respectively (table 1). It should be noted, that the *ey* expression detected in the embryo at this stage is relatively diffuse and weak, in agreement with previous *in situ* studies in which *ey* expression was not detected until stage 10 [11]. In Kc cells, the expression of *toy* correlates with modest expression of the genes immediately downstream in the RD pathway, *so* and *eya* (figure 1). In the embryo, the expression patterns for *toy, so* and *eya* all extensively overlap in the characteristic anterior dorsal band (figure 5), supporting a role for TOY in activating these downstream target genes. Furthermore, *nmo*, which is present at high levels in Kc (6.87 TPM) and S2 (8.94 TPM) cells, is also expressed in this same anterior band (and also in more posterior locations), indicating that the NMO protein kinase may also play a role in establishing the early activation of the other components of the RDGN in the embryo (figure 5). Despite these potential activation events, it is clear that TOY is not sufficient to activate expression of *ey* in Kc cells. The absence of *ey* expression may be explained in part by the very low level expression of *hth* (0.06 TPM) and *tsh* (0.17 TPM), both of which show an overlapping pattern of expression in stage 6 embryos (figure 5) and have previously been shown to play a context-dependent role in activating *ey* [29–31]. However, it may also indicate that additional unknown components of the network are capable of repressing *ey* expression in Kc cells.

In S2 cells, the transcriptional landscape of the RDGN appears to be dominated by the expression of *ey and tsh* (figure 1). These two genes demonstrate largely independent patterns of expression in stage 6 embryos (figure 5) and act antagonistically on the immediate downstream components of the RD network, *so* and *eya* [30]. The very high level of *tsh* expression in S2 cells (62.22 TPM) appears to be sufficient to completely repress expression of *so* and *eya*, resulting in a suppression of the retinal determination pathway. In addition, the expression pattern of *tsh* in the embryo shows no overlap with either *so* or *eya* (figure 5), supporting a repressive function. Mutually exclusive patterns of expression for *tsh* and *tiptop* (*tio*) are also observed in the stage 6 embryos (figure 5). Given that these two genes encode structurally similar C2H2 zinc finger TFs [32], demonstrate functional redundancy in the RDGN, and act as transcriptional repressors of each other [29], it is perhaps surprising that higher levels of *tio* (0.46 TPM) are not detected in Kc cells along with the low level of *tsh* present. This result may be indicative of a regulatory role for additional previously uncharacterized components of the RD network in the *Drosophila* cells.

### 2.5. Potential novel RDGN components

Combining our PPI analysis with the transcriptome expression data in the two cell lines, reveals 16 new genes as potential components in the RDGN. All 16 genes have at least 14 node connections within the *ey* and/or *toy* PPI networks and have an expression read count of 8 or above, equating to a TPM score of 0.15 or above, in at least one of the cell lines (table 4). Eleven of the 16 genes encode TFs (*pan*, *fkh*, *cad*, *Sox100B*, *SoxN*, *nau*, *Mad*, *pnr*, *ci*, *exd* and *foxo*), one encodes a Wnt signalling ligand (*Wnt5*), one for the CBP histone acetyltransferase (*nej*), one encodes a FGF ligand (*bnl*), one encodes a cadherin active in the JAK-STAT signalling pathway (*shg*) and one encodes a receptor in the HH signalling pathway (*smo*). Many of these identified genes are highly pleiotropic and therefore are likely to play functional roles within additional gene regulatory networks during embryogenesis. Indeed, their activity in the RDGN and beyond may indicate their central importance in fundamental molecular mechanisms in parallel cell determination pathways.

**Table 4.** Potential novel components of the RDGN. The 16 genes we identified as potentially novel components of the RDGN, along with *eyeless* and *twin of eyeless*, are listed with the number of node connections in each PPI network, and the TPM values and log_2_ expression ratio in Kc and S2 cell lines. The functional activity of the protein encoded by each gene is also shown.

gene	symbol	gene ID	ey node conn	toy node conn	KC TPM	S2 TPM	log2 (KC/S2)	notes
*eyeless*	ey	43812	99	58	0.05	2.8	−5.80	paired homeobox TF
*twin of eyeless*	toy	43833	58	61	21.78	0.19	6.83	paired homeobox TF
*Wnt oncogene analog 5*	Wnt5	32838	40	32	24.58	16.25	0.57	Wnt signalling ligand
*pangolin*	pan	43769	30	22	21.99	25.51	−0.24	HMG box TF, Wg signalling pathway
*fork head*	fkh	43383	29	23	0.15	0	3.58	fork head (winged helix) box TF
*caudal*	cad	35341	0	29	1.36	0.03	5.74	homeobox TF
*Sox100B*	Sox100B	45039	25	25	0.04	1.46	−5.27	E Sox domain TF
*SoxNeuro*	SoxN	44275	23	22	0.77	0.05	3.91	HMG box TF
*nautilus*	nau	42799	23	19	0	0.43	−5.09	bHLH TF
nejire	nej	43856	23	0	15.25	19.36	−0.37	CBP, histone acetyltransferase
*mothers against dpp*	Mad	33529	21	0	14.82	47.07	−1.69	Mad domain TF, BMP signalling pathway
*branchless*	bnl	42356	21	20	45.35	1.23	5.18	fibroblast growth factor (FGF) ligand
*shotgun*	shg	37386	21	0	2.57	3.66	−0.54	cadherin, JAK-STAT signalling pathway
*pannier*	pnr	44849	20	20	1.41	221.13	−7.32	GATA TF
*cubitus interruptus*	ci	43767	17	0	1.69	0.3	2.46	C2H2 ZF TF, hh signalling pathway
*smoothened*	smo	33196	15	11	8.34	13.02	−0.67	Smo-type receptor, hh signalling pathway
*extradenticle*	exd	32567	14	0	63.15	90.49	−0.55	TALE Homeobox TF
*fork head box O*	foxo	41709	14	0	24.84	9.28	1.39	fork head (winged helix) box TF

A detailed search of the literature reveals that while none of the 16 have been formally assigned as members of the RDGN, there is evidence that three of them (*cad*, *Mad* and *exd*) do play a role in regulating the network. The regulatory role for the gap CAD homeobox TF as a transcriptional repressor of *toy* in the posterior of the early embryo is described earlier in this paper (see §2.3 for details) and in our prior study examining the *cis*-regulatory enhancers that drive *toy* expression in stage 5 embryos [13]. MAD is the primary TF that mediates the cellular response to BMP-like ligands, including DPP. Upon phosphorylation, MAD forms a complex with MEDEA and translocates to the nucleus where, in conjunction with cofactors, it regulates expression of BMP response target genes. Intriguingly, MAD is phosphorylated by the NMO protein kinase [33]. As *nmo* is already known to genetically interact with *ey* and *eya* in the RDGN [34,35], it would suggest that NMO is in fact capable of phosphorylating multiple target substrates within the network. The EXD protein is a TALE homeobox TF that is imported into the nucleus upon binding of HTH (which is itself also a TALE homeobox TF), in a process that is downstream of DPP and WNT signalling [36]. Once in the nucleus, the HTH-EXD complex functions as a cofactor that can modulate the DNA-binding specificity of Hox TFs [37], but also have additional Hox-independent functions that promote cell division in the undifferentiated eye field. An important role for this complex in the RDGN was revealed in loss-of-function experiments, in which mutations in either protein led to the formation of ectopic eyes [38,39]. This result indicates that the normal function of the HTH-EXD complex is to suppress eye development, likely through the direct repression of RD genes including *eya* and *dac* [38].

**Figure 6.**
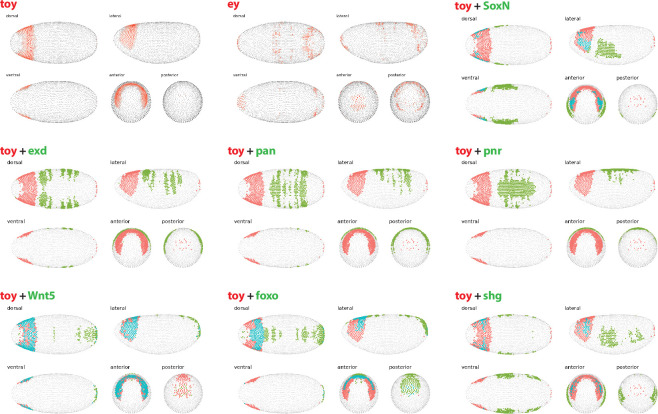
Embryonic expression patterns of select novel RDGN components. The expression of *twin of eyeless* (*toy*) and *eyeless* (*ey*) in stage 6 embryos is shown. The expression of *toy* with seven other genes identified as potential novel components of the RDGN is also shown in each panel. In each case, the genes and their expression in stage 6 embryos are colour coded with *toy* in red, the second gene in green and any overlapping expression shown in blue. All data generated from DVEX platform as described in §4.

Of the 16 potentially novel RDGN we have identified, only *Sox100B* and *nau* do not have characterized expression patterns in stage 6 embryos [28], with the remaining 14 genes demonstrating a range of distinct expression patterns (electronic supplementary material, figure S2). Many of the genes, including *exd*, *pan* and *pnr*, display patterns of expression that are restricted predominantly to the posterior of the embryo and therefore do not overlap extensively with the anterior dorsal band of *toy* expression, potentially indicating a repressive role for these TFs (figure 6). Supporting this interpretation is the fact that all three are expressed at lower levels in Kc cells when compared with S2 cells, significantly so in the case of *pnr* (table 4). PNR is a GATA TF that acts as a regulator of proneural ACHAETE-SCUTE complex genes [40]. Its currently characterized roles include dorsal cell fate determination and regulation of a variety of developmental systems including heart and neuronal tissues [41], but the massively higher level of expression in S2 cells (221.13 TPM) when compared to Kc cells (1.41 TPM) may be indicative of a potential role in repressing *toy* transcription and/or activating *ey*. PAN is an HMG-domain TF that is a key component of the canonical WG signalling pathway and demonstrates a bimodal function, as it can act as a transcriptional repressor (when bound to GRO) or activator (when bound to ARMADILLO) to promote cell fate specification [42].

By contrast, a subset of the 16 novel RD genes, including *Wnt5*, *foxo* and *shg*, are expressed in patterns that extensively overlap with *toy* expression in stage 6 embryos (figure 6). These overlapping expression patterns may be indicative of positive regulatory interactions between the genes, which is supported by the fact that *Wnt5* and *foxo*, along with *toy*, are expressed at higher levels in Kc cells than S2 cells (table 4). WNT5 is extensively studied and is known to act as a signalling ligand for the RYK family of receptor tyrosine kinase-related WNT receptors in multiple different developmental process, including embryonic axon guidance and antennal lobe patterning [43,44]. The very high level of connectivity of WNT5 in both of the PPI networks we analysed (it ranks third in both with 40 and 32 node connections, behind only *ey* and *toy*) and its high level of expression in both cell types (24.58 TPM in Kc, 16.25 TPM in S2) strongly suggest a potential additional role for WNT5-mediated signalling in the RDGN. The FOXO fork head box TF has previously been shown to be involved in the regulation of the insulin signalling pathway [45], but the extensive overlap in expression pattern with *toy* in the anterior dorsal region of the embryo (figure 6) and the significantly higher level of expression in Kc cells (24.84 TPM) than S2 cells (9.28 TPM) indicate a potential role in the very earliest regulatory steps of the activation of the RDGN. SHG is an E-cadherin with a characterized positive regulatory role in the JAK-STAT signalling pathway [46], but no known connection to the RDGN.

Finally, *SoxN*, which encodes an HMG-domain TF, displays an interesting partially overlapping pattern of expression with *toy* in stage 6 embryos (figure 6), which may suggest a concentration-dependent regulatory interaction between these two factors. In early embryos, SOXN is known to specify neural progenitors in the central nervous system, while in later embryos it negatively regulates WG signalling and controls expression of genes required for denticle construction [47,48]. SOXN demonstrates a very high level of connectivity in both of our PPI networks (it ranks seventh with 22 node connections in both networks) and, while expressed at only moderate levels, it is significantly higher in Kc cells (0.77 TPM) when compared to S2 cells (0.05 TPM).

### 2.6. Novel TF binding at enhancers for genes in the RDGN

**Figure 7.**
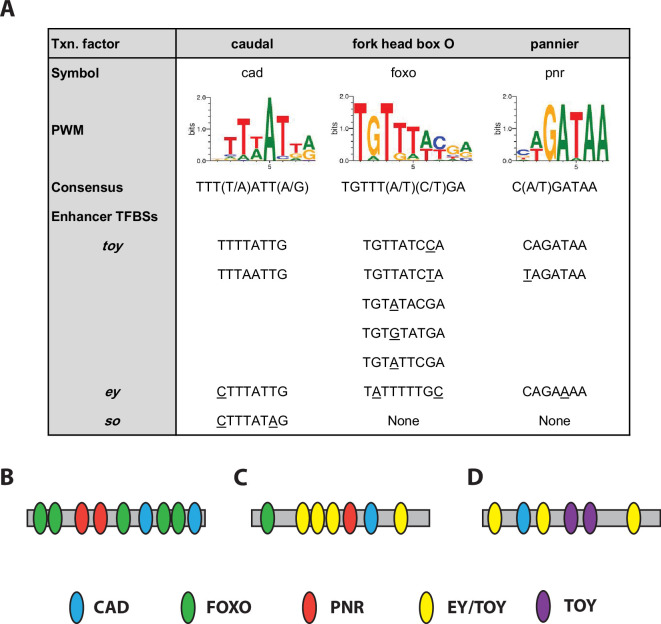
TF binding sites in the enhancers for genes in the retinal determination network. (A) Predicted binding sites for CAUDAL, FOXO and PNR. The position weight matrix (PWM) and consensus binding site for each TF is shown, along with bioinformatically predicted binding sites in the previously characterized minimal enhancers for the core RDGN genes; *twin of eyeless* (*toy*), *eyeless* (*ey*) and *sine oculis* (*so*). Nucleotides in binding sites that differ from the consensus sequence for each TF are underlined. The physical organization of the binding sites on the minimal enhancers for *toy* (B), *ey* (C) and *so* (D) is also shown with the TF binding sites colour coded as shown in the key.

To further investigate the potential regulatory activity for the novel TF components of the RDGN identified in this study, we analysed the predicted binding of CAD, FOXO and PNR, each of which has well-characterized binding site data [49], in the previously characterized enhancers that control expression of *toy* [13], *ey* [50] and *so* [7] in the embryo. In each case, we used bioinformatic tools to search the minimal identified enhancer region for predicted high affinity binding sites (figure 7A). The 446 bp signature motif region from the *twin of eyeless zone 2* enhancer [13] harbours two CAD sites, five FOXO sites and two PNR sites (figure 7B). The 280 bp *eyeless intron 1 eye-specific* enhancer [50] contains one predicted site each for CAD, FOXO and PNR, along with the four previously characterized binding sites that can bind either of the PAX6 paralogues (TOY and EY) [15] (figure 7C). The 428 bp *sine oculis so10* enhancer contains a single predicted site for CAD, along with the three previously characterized binding sites that can recruit either of the PAX6 paralogues and two binding sites shown to be specific for TOY [7], but no predicted binding sites for FOXO or PNR (figure 7C).

Support for the specific enrichment of the TF binding sites in the enhancers is provided by analysis of their frequency in the *toy* enhancer when compared to the neighbouring 446 bp 3′ genomic region or 100 randomly scrambled sequences generated from the 446 bp enhancer. In the case of FOXO, the enhancer contains five predicted sites, while the adjacent genomic region has two and the scrambled sequences contain 1.584 sites on average. For PNR, the enhancer contains two predicted sites, the adjacent genomic region has zero and the scrambled sequences contain 1.008 sites on average. For CAD, the enhancer and the adjacent genomic region both contain two predicted sites, while the scrambled sequences contain 1.958 sites on average. However, it should be noted when restricting the analysis to only include high stringency CAD binding sites, achieved by applying a 25^th^ percentile cutoff (see §4 for details), the enhancer still contains the same two predicted sites, but the adjacent genomic region has zero and the scrambled sequences contain only 0.577 sites on average. The existence of strong predicted binding sites for the three TFs in these critical enhancer *cis*-regulatory modules, particularly for *toy* as the master regulator of the initiation of the RD pathway in early embryogenesis, lends significant support to the idea that CAD, FOXO and PNR are important players in the regulation of the RDGN.

## 3. Conclusions

Integrating our analysis of the transcriptional profiles in Kc and S2 cells, the interaction networks for the *eyeless* (*ey*) and *twin of eyeless* (*toy*) genes, and expression patterns for genes in the early *Drosophila* embryo enabled us to achieve three major goals. Firstly, the data confirm that the regulatory logic of the 21 previously identified components of the retinal determination gene network (RDGN) is essentially intact in the two *Drosophila* cell lines. While each cell line expresses a high level of just one of the *Pax6* paralogues (*toy* in Kc cells, *ey* in S2 cells), this is not sufficient to extensively activate the downstream components of the core RD pathway and ultimately neither cell type adopts an eye-like fate. In S2 cells, suppression of the pathway appears to be controlled by very high level expression of the *teashirt* (*tsh*) transcription factor acting to downregulate *sine oculis* (*so*) and *eyes absent* (*eya*). In Kc cells, the mechanism of repression is less clear. However, our second major finding is that both *ey* and *toy* demonstrate extensive interactions with over 100 genes previously not known to be involved in the RDGN. If we only consider genes that show higher levels of connectivity in the network (14 or above PPI node connections) and are expressed at a level above 0.15 TPM in one of the two cell types, this allows us to identify 16 candidates as novel components of the RDGN. Finally, we were able to analyse the expression patterns of these novel components, along with the previously known RDGN members, in embryos and ascertain their potential for activating or repressing roles in the network. Eleven of the 16 novel genes encode transcription factors. Analysis of the binding sites for three of these (*caudal* (*cad*), *fork head box O* (*foxo*) and *pannier* (*pnr*)) in characterized enhancers for *toy*, *ey* and *so* confirms the potential for transcriptional regulatory activities in the earliest stages of the RDGN. Detailed characterization of the molecular mechanisms of the regulatory contribution of the pre-existing and novel RDGN components we identified, including RNAi-mediated knock down of the genes in the *Drosophila* cell lines, should be a high priority in future studies.

## 4. Material and methods

### 4.1. Cell culture and RNA isolation

The Kc167 (Kc, RRID: CVCL_Z833) and S2-DRSC (S2, RRID: CVCL_Z992) cell lines used in this study were obtained from the *Drosophila* Genomics Resource Center (DGRC). Cells were thawed, passaged and frozen as previously described [21]. Cells were harvested at approximately 5 × 10^6^ cells ml^−1^ density from six replicate samples grown in 25 cm^2^ canted neck culture flasks (Corning) and RNA isolated as previously described using a RNeasy kit following the manufacturer’s protocol (Qiagen) [21].

### 4.2. RNA sequencing and read mapping

Library construction and sequencing were performed at the Beijing Genomics Institute. Briefly, 10 µg of total RNA was enriched for poly(A)^+^RNA by oligo(dT) selection and used to generate a cDNA library for sequencing, as previously described [21]. The libraries were sequenced on the Illumina nanoball (DNBSEQ) PE100 platform. Sequencing data was filtered to remove reads that contained adaptor sequences, reads whose N content was greater than 5%, and low-quality reads (quality score less than 15 for 20% or greater of the total bases in the given read). The generated clean read fastq files were aligned using Bowtie2 software to the *Drosophila melanogaster* genome (Release 6 plus ISO1 mitochondrial, RefSeq accession: GCF_000001215.4) and used to calculate quantitative TPM scores as previously described [21].

### 4.3. Gene expression analysis

Manual curation was employed to identify a list of 21 genes known to be components of the retinal determination gene network (RDGN) in *Drosophila melanogaster* (figure 1) [1,2,10]. Expression in embryos was visualized at single cell resolution using the *Drosophil*a Virtual Expression eXplorer (DVEX) platform at the 0.9 threshold for normalized expression level [28]. Three-dimensional gene expression patterns in cellularized stage 6 embryos and the two-dimensional t-SNE representation of the 11 major cell clusters grouped by transcriptome similarity were analysed.

### 4.4. Protein–protein interactions

The STRING database (http://string-db.org/) [51] was used to evaluate the protein–protein interaction (PPI) network of the genes in the Dr. Tom package (http://biosys.bgi.com), essentially as previously described [22]. All the nodes in the PPI network were annotated genes (mRNAs) in the *Drosophila melanogaster* genome (Release 6 plus ISO1 mitochondrial, RefSeq accession: GCF_000001215.4), with either *eyeless* or *twin of eyeless* selected as the initial seed protein. Minimum PPI score was set to 500, with no limit on the maximum number of interactions. This enabled the identification of all the partners in each PPI network with a high confidence for physical interaction in STRING11.5. In the case of *eyeless*, a total of 99 nodes were identified (figure 2A) and for *twin of eyeless*, 61 nodes were identified (figure 2B).

### 4.5. Transcription factor binding site predictions

Predictions were performed on genomic regions previously identified as minimal enhancers for *twin of eyeless* (446 bp signature motif of zone 2 enhancer [13]), *eyeless* (280 bp eye-specific enhancer, RedFly ID: RFRC:0000000285.003 [50]) and *sine oculis* (428 bp so10 enhancer, RedFly ID: RFRC:0000000486.003 [7]). For each enhancer, binding sites were predicted using the MAST algorithm [52] for CAUDAL (CAD), FORK HEAD BOX O (FOXO) and PANNIER (PNR). The aligned sequences used to construct the position weight matrix (PWM) for each TF in this analysis were downloaded from the FlyFactorSurvey database [49]. All sequences were obtained from a bacterial one-hybrid system, with sequences for CAD (1420 total sequences) sequenced using SOLEXA and sequences for FOXO (20 total sequences) and PNR (17 total sequences) sequenced using standard Sanger method.

Binding sites were identified on each enhancer using threshold scores that corresponded to the known binding sequences used to build the PWMs and reflecting the difference in quality and quantity of sequences obtained using SOLEXA versus Sanger sequencing approaches. In the case of CAD, a 50th percentile cutoff was used. For FOXO and PNR, a 100th percentile cutoff was used. To analyse the enrichment of binding sites in the 446 bp *toy* enhancer, two different control sequences were utilized. The first was the adjacent 446 bp genomic region immediately 3′ of the defined signature motif enhancer [13]. The second was obtained by randomly scrambling the 446 bp enhancer sequence and then scanning for predicted binding sites. The random scrambling was repeated a total of 100 independent times and the average number of identified binding sites for each TF recorded.

## Data Availability

The datasets supporting the results of this article are available at the NCBI Sequence Read Archive (SRA) under BioProject accession number PRJNA937779. Supplementary material is available online [53].
